# Clinical Olsen Grading Does Not Reflect Basal Growth in Actinic Keratoses: Two-Center Retrospective Analysis

**DOI:** 10.3390/cancers17233794

**Published:** 2025-11-27

**Authors:** Lutz Schmitz, Conrad Falkenberg, Julius Balkenhol, Paul Melzer, Wolfgang G. Philipp-Dormston, Thomas Dirschka

**Affiliations:** 1 CentroDerm, Heinz-Fangman-Straße 57, 42287 Wuppertal, Germanyj.balkenhol@centroderm.de (J.B.);; 2 Department of Dermatology, Venereology and Allergology, Ruhr-University, 44780 Bochum, Germany; 3 Faculty of Health, University of Witten-Herdecke, Alfred-Herrhausen-Straße 50, 58448 Witten, Germany; 4 Hautzentrum Köln, 50996 Cologne, Germany

**Keywords:** actinic keratosis, basal proliferation, histopathology, risk stratification

## Abstract

**Background/Objectives**: Actinic keratosis (AK) is a precancerous lesion that may progress into cutaneous cancer. However, the progression potential of individual lesions remains unpredictable. The histological basal proliferation pattern of AKs may serve as a risk marker for progression, yet it cannot be assessed clinically. The objective was to evaluate whether Olsen grading (hyperkeratosis of AKs) and pain as clinical markers correlate with the histological basal proliferation (PRO) and different histological aspects. **Methods**: In this retrospective two-center study, 380 clinically diagnosed AKs were graded according to the clinical Olsen classification (I–III) and assessed for pain upon palpation. Histologically, they were classified based on their basal- (PRO I–III) and upward-directed (AK I–III) growth patterns, and additional histopathological features, such as acantholysis, were documented. **Results**: Olsen grading showed weak correlation with the PRO classification (Spearman’s rho = 0.136, *p* = 0.008), with exact agreement of 36.3% (κ = 0.07). Pain was significantly associated with higher PRO grades (*p* = 0.005) and acantholysis (*p* = 0.023) but not with Olsen grades or upward-directed growth (AK I–III). **Conclusions**: Olsen grading does not allow reliable prediction of basal proliferation patterns in AKs. Its use as a clinical severity scale may suggest progression relevance; however, no substantiated association with histological indicators of transformation could be demonstrated in this study. The presence of pain, however, correlated with high PRO grades and the presence of acantholysis.

## Simple Summary

Actinic keratosis is a common sun-damaged skin spot that can develop into cancer, but the risk for any single lesion is hard to judge. In practice, doctors grade these spots by how thick and scaly they appear (Olsen grade) and often treat thicker ones more aggressively. We asked whether this visible thickness reflects the pattern of cell growth at the base of the lesion seen under the microscope. This feature is thought to signal a higher risk of progression. We reviewed 380 lesions from two centers, comparing the clinical thickness grade with the microscopic growth pattern, and also noted whether touching the lesion caused pain. Visible thickness matched the basal growth pattern only weakly. Pain was linked to more active basal growth, while thickness and upward growth patterns were not. These findings suggest that thickness alone should not guide risk judgments and that symptoms such as pain should be considered alongside approaches to evaluate basal growth even before cutting out the lesion.

## 1. Introduction

Actinic keratoses (AKs) are among the most frequent precancerous skin lesions in fair-skinned individuals and reflect cumulative ultraviolet (UV) damage. They are currently defined as part of a broader spectrum of UV-induced epithelial dysplasia, which includes field cancerization and in situ carcinoma, as outlined in the most recent European consensus guideline [1]. Their prevalence is estimated at 14% globally and continues to rise due to population ageing and increasing recreational sun exposure [2,3,4]. The potential of AKs to progress into invasive cutaneous squamous cell carcinoma (cSCC) is well established [5]. A ten-year cumulative risk of 17.1% has been reported following AK diagnosis, compared to 5.7% in matched controls [6]. Risk is further increased in older patients, men, and immunosuppressed individuals [7,8,9]. In addition, several clinical characteristics such as lesion location (particularly on the scalp, ears, and lips), lesion multiplicity, and high Actinic Keratosis Area and Severity Index (AKASI) scores have been associated with increased progression risk [10,11]. Individuals with a prior diagnosis of cSCC in situ show a more than 16-fold elevated risk of developing invasive cSCC within one year compared to the general population [12]. Despite these known risk factors, reliable prediction of the progression of individual lesions remains challenging. Several classification systems and other parameters for AK exist in the clinical and histological domains. In the clinical setting, AK is classified into three grades according to Olsen et al. (I-III) based on palpability and the degree of hyperkeratosis [13,14]. The Olsen classification is considered the gold standard for the clinical assessment of actinic keratoses today. Another clinical parameter is pain upon palpation, linked to AK and cSCC [15,16,17]. Histopathological classification systems and definition of AK variants have been developed to stratify AKs based on histomorphological features. Röwert-Huber et al. described the degree of upward-directed proliferation of atypical keratinocytes across three grades (AK I–III) [18]. Schmitz et al. introduced the PRO classification to better capture basal proliferation patterns, categorising AKs into PRO I–III [19]. PRO describes the downward-directed extension of atypical keratinocytes. Contrary to earlier assumptions, later studies indicate that lesions with atypia confined to the basal layer (AK I) may carry the highest risk of progression [20,21]. Higher PRO grades, particularly PRO III, have been associated with histological characteristics of invasive cSCC and may reflect an increased progression risk [19,21]. This interpretation is supported by both European and national guidelines, which consider the PRO classification a potential indicator of progression risk in AKs [1,22]. Low Röwert-Huber classification (AK I) and high PRO classification (PRO III) combined were associated with the highest risk of cSCC [20,21,23]. Many histomorphological variants of AK exist, of which, in total, six should be reported upon presence according to national guidelines [22]. Acantholysis as one variant and high PRO grades (PRO III) were linked to a higher risk of cSCC in organ transplant patients [23]. Despite the presence of histological risk stratification, their applicability in clinical routine is limited since AK is not routinely biopsied, and excision or biopsy is not the first-line approach [22]. Therefore, correlations of clinical and histological classifications and parameters are to be determined. Previous works could not find an association between the histological Röwert-Huber and clinical Olsen classifications [24]. While another work has linked high PRO grades, acantholysis, and pain to treatment-resistant AK, the association between clinical and histological parameters was missing [17]. The association between clinical classification and parameter OLSEN and pain with the histologic PRO classification and AK variants has not been performed. The aim of this study was therefore to investigate whether clinical grading according to the Olsen system and the presence of pain allow prediction of the histological basal growth pattern or the presence of variants in AKs and thus can reflect biologically relevant features associated with progression risk.

## 2. Materials and Methods

### 2.1. Study Design and Tissue Collection

This retrospective observational study was conducted at two dermatology centers in Germany: Haut Zentrum (Cologne) and CentroDerm (Wuppertal). Biopsies were performed for medically indicated diagnostic or therapeutic purposes and were not conducted solely for research. Lesions were located on chronically sun-exposed skin, including the balding scalp, forehead, and face. Punch biopsies either included the whole area of the lesion, or a full-excision biopsy was performed. Prior to biopsy, each lesion was clinically graded using the Olsen classification system, which differentiates AKs based on palpability and visual presentation. Grade 1 lesions were only slightly palpable and better felt than seen; grade 2 lesions were moderately thick, clearly visible, and palpable; and grade 3 lesions were very thick, hyperkeratotic, and clinically obvious. In addition, lesions were gently manipulated by placing the fingertip on the lesion, increasing pressure for two seconds, and performing slight lateral rotation, to determine whether mechanical stimulation caused pain, which was documented as part of the clinical evaluation. Tissue biopsies of both centers were routinely processed and analyzed in one histopathology lab CentroDerm (Wuppertal). The study was approved by the Ethics Committee of Witten/Herdecke University (251/2023) and conducted in accordance with the principles outlined in the Declaration of Helsinki.

### 2.2. Histological Assessment

Biopsied specimens were fixed in 10% neutral buffered formalin, processed using standard histological techniques, and stained with hematoxylin and eosin (H&E). Only tissue samples with a minimum diameter of 3 mm and an intact, evaluable dermo-epidermal junction were included. Specimens were excluded if they lacked histological evidence of AK, showed signs of invasive growth, or were insufficient in size or quality. Standard histological procedures routinely applied in dermatopathology were used to establish the final diagnosis of AK, including deeper sections when necessary to exclude cSCC. Routine histological analysis was performed independently by two board-certified dermatopathologists (LS, CF) who were blinded to the clinical grading. In cases of discordant evaluation, consensus was reached by joint assessment using a multi-headed microscope. Each lesion was classified according to two established histological systems. The first, introduced by Röwert-Huber et al., categorizes lesions based on the vertical extent of atypical keratinocytes: AK I shows atypia confined to the lower third of the epidermis, AK II involves up to two-thirds, and AK III features full-thickness atypia. The second system, proposed by Schmitz et al., focuses on basal growth patterns along the basement membrane. In PRO I (crowding), densely packed atypical basal keratinocytes produce a basophilic appearance without architectural distortion. In PRO II (budding), small hemispheric nests of atypical keratinocytes protrude into the papillary dermis, remaining thinner than the overlying epidermis. PRO III (papillary sprouting) is characterized by spiky or filiform projections of atypical keratinocytes extending into the upper dermis, exceeding the thickness of the epidermis above. Where applicable, additional histological features were documented, including acantholysis, atrophy, Bowenoid changes, hyperkeratosis, and lichenoid inflammation.

### 2.3. Statistical Analysis

Statistical analysis was carried out using IBM SPSS Statistics Version 29. Cases were considered “matched” when the clinical Olsen grade numerically corresponded to the histological classification (e.g., Olsen grade 2 matching AK II or PRO II). All other combinations were considered “non-matched” cases. Spearman’s rank correlation coefficient (*ρ*) was calculated to assess the association between clinical and histological grading systems. Statistical significance was defined as *p* < 0.05. Agreement beyond chance was further evaluated using Cohen’s kappa coefficient (*κ*). In non-matched cases, whether the clinical grade underestimated or overestimated the histological grade was recorded. The distribution of matched and non-matched cases was analyzed accordingly. The association between pain response and each grading system (Olsen, AK, and PRO) was also evaluated. In a subset of 274 cases, additional histological features were tested for correlation with pain using Fisher’s exact test. For the PRO classification, which comprises three ordinal categories, pairwise comparisons were performed between subgroups when overall significance was observed.

## 3. Results

A total of 380 clinically diagnosed AKs were included in the study. The mean patient age was 74.1 years. Clinically, 30.0% of lesions were classified as Olsen grade 1, 51.1% as grade 2, and 18.9% as grade 3 (Table 1).

**Figure 1:**
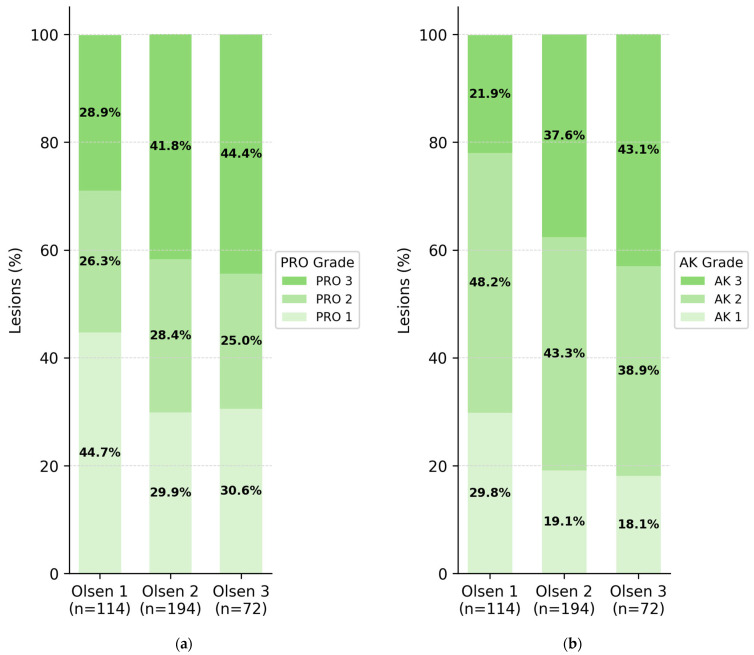
Stacked bar charts show the relative frequency of histological classifications according to PRO (**a**) and AK (**b**) systems, stratified by clinical Olsen grades 1–3. (**a**) In the PRO classification, the proportion of PRO III lesions increases with clinical severity (28.9% in Olsen 1, 41.8% in Olsen 2, and 44.4% in Olsen 3), yet the overall correlation remains weak (Spearman’s *ρ* = 0.136; *p* = 0.008; agreement 36.3%; κ = 0.07). (**b**) Similarly, in the AK classification, the percentage of AK III lesions rises with increasing Olsen grade (21.9%, 37.6%, 43.1%), but substantial overlap persists (*ρ* = 0.170; *p* = 0.001; agreement 39.2%; κ = 0.06). These findings illustrate that Olsen grading does not allow reliable prediction of either basal proliferation (PRO) or vertical dysplasia (AK) patterns in actinic keratoses.

Histologically, 34.5% of lesions were PRO I, 27.1% PRO II, and 38.4% PRO III, while the vertical AK classification yielded 22.1% AK I, 43.9% AK II, and 33.9% AK III. Both histopathological classifications (PRO and AK) were independently assessed by two investigators. Interrater reliability between them was evaluated using weighted Cohen’s kappa, demonstrating almost perfect agreement for both PRO (κ = 0.81, 95% CI 0.72–0.86) and AK grading (κ = 0.85, 95% CI 0.79–0.91). When comparing Olsen grading with the histological Röwert-Huber classification, a statistically significant but weak correlation was observed (Spearman’s *rho* = 0.170, *p* = 0.001), with exact agreement in only 39.2% of cases and a Cohen’s *κ* of 0.06 (Figure 1b). A similar result was obtained for the PRO classification, where correlation with Olsen was again weak (*ρ* = 0.136, *p* = 0.008), with an agreement rate of 36.3% and *κ* = 0.07 (Figure 1a).

**Figure 2:**
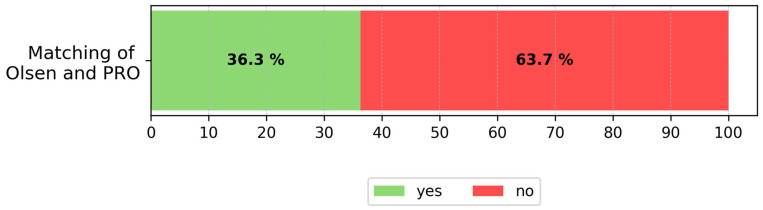
Overall agreement of clinical Olsen and histological PRO classification. The bar illustrates overall agreement between Olsen clinical grading and histological PRO classification. Exact correspondence was found in only 36.3% of cases, while 63.7% of cases showed mismatch.

The distribution of histological subtypes across Olsen grades showed considerable overlap. Notably, even among Olsen grade 1 lesions, 21.9% were classified as AK III and 28.9% as PRO III. These findings demonstrate that clinical grading using the Olsen system is not a reliable indicator of histological severity in AKs—neither in terms of vertical atypia (AK classification) nor basal proliferation pattern (PRO classification). To further explore these discrepancies, Figure 2 summarizes the overall agreement versus the mismatch between Olsen and PRO classifications.

**Figure 3:**
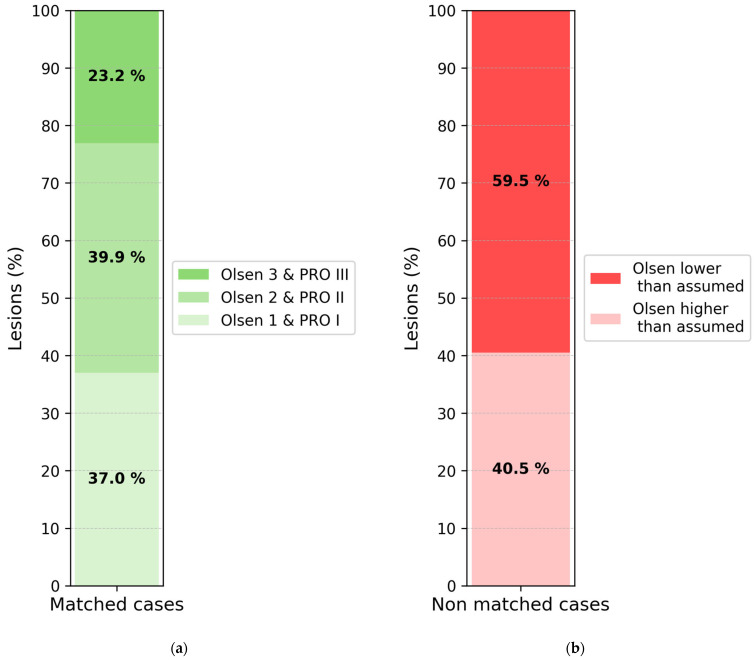
Clinical Olsen and histological PRO classification: Distributions of lesions with matching grades and distribution of non-matched cases. (**a**) The left panel shows the distribution of histological PRO subtypes within matched cases. Notably, even among these, 23.2% were PRO III, indicating that high-risk basal proliferation patterns can occur despite apparent clinical–histological agreement. (**b**) The right panel depicts the direction of mismatch among non-matched cases: 59.5% were underestimated clinically, while 40.5% were overestimated. This pattern suggests a tendency of the Olsen grading to underestimate histological severity.

Among the 63.7% of non-matching cases, a directional analysis revealed that the clinical grade underestimated histological severity in 59.5% of lesions (Figure 3b). Importantly, even within the 36.3% of matched cases, nearly one quarter (23.2%) were histologically graded as PRO III, indicating that high-risk basal proliferation patterns may be missed even when clinical and histological scores appear numerically aligned (Figure 3a).

When comparing the Olsen grade to the presence of histomorphological variants, there was no significant correlation for any variant but hyperkeratotic AK (*p* < 0.001; Table 2). In particular, OLSEN III lesions were significantly more hyperkeratotic than OLSEN I and OLSEN II lesions, while there was no significant difference between OLSEN I and OLSEN II.

Pain upon scratching with a finger was reported in 127 of 380 lesions (33.4%). No significant association was found between pain and Olsen grade (*p* < 0.690) or vertical AK classification (*p* < 0.115). However, pain was significantly associated with the PRO classification (*p* < 0.005), with the highest frequency observed in PRO III lesions (41.1%) and a significant pairwise difference between PRO III and PRO I (*p* < 0.001; Table 3). In the subset of lesions with additional histological analysis (*n* = 274), the presence of acantholysis was also significantly associated with pain (*p* = 0.023). No significant associations were observed between pain and atrophy, Bowenoid changes, hyperkeratosis, or lichenoid infiltrates.

## 4. Discussion

This study aimed to investigate whether the clinical Olsen classification of AKs and the presence of pain correlate with their histological basal proliferation pattern, as defined by the PRO classification, and histomorphological variants. The clinical Olsen grading was not predictive of the underlying histological basal growth behavior, with a correlation coefficient of *ρ* = 0.136 and a low level of exact agreement (κ = 0.07). These findings complete the previous reports by Schmitz et al., stating no correlation between clinical and histological classifications, who previously found limited correlation between Olsen grades and the upward-directed extension according to Röwert-Huber. It underlines the restricted interpretability of Olsen grading in terms of histological architecture [24]. The histological basal proliferation pattern, particularly PRO III, has been proposed as a surrogate marker for progression risk [22]. Prior studies have demonstrated that sprouting basal growth correlates with biological aggressiveness and is more frequently associated with dermal invasion [17,21]. In our study, PRO III lesions were significantly associated with the presence of pain. Although pain is commonly associated with invasive cSCC, recent studies have suggested that it may also occur in biologically active actinic keratoses. Pain has been identified as a predictor of cSCC in immunosuppressed patients [15] and is more frequently reported in cSCC than in AKs [16]. In cSCC, the presence of pain was associated with the histologic presence of inflammatory infiltrates and the presence of neutrophils [25]. Additionally, it has been described in treatment-resistant AKs, which are considered to show features of early progression [17]. In our cohort, pain correlated with PRO III lesions, but not with Olsen grade or vertical dysplasia, supporting its potential role as a clinical indicator of biologically active lesion states. These findings align with previous reports linking pain in cSCC to adverse histological parameters such as tumor depth, poor differentiation, perineural invasion, and acantholysis [26]. While such invasive features were not evaluated in our study, the observed association between pain and basal proliferation pattern may indicate increased malignant potential, already at the in situ level. Furthermore, the presence of pain was significantly associated with the presence of acantholysis. Acantholysis has been associated with a higher risk of cSCC in otherwise healthy and organ transplant patients [20,23]. Acantholysis is characterized by loss of intercellular adhesion, one critical and established concept in carcinogenesis [27]. However, there is currently no evidence of the loss of intercellular adhesion in AK. In synopsis, while Olsen does not correlate with any histological feature but hyperkeratotic AK, the presence of pain correlates with established histological parameters associated with risk of invasion including high basal proliferation and acantholysis. These results suggest that clinically assessed lesion severity, as indicated by Olsen grade, does not reflect biologically relevant histopathological characteristics. This observation is particularly relevant considering studies that have associated higher Olsen grades, especially grade III, with an increased risk of progression to invasive cSCC [28]. In a secondary analysis of a randomized clinical trial, Olsen grade III lesions were reported to carry a 20.9% four-year risk of developing cSCC [29]. However, it remains uncertain whether these lesions truly represent a transformation from in situ disease or were invasive from the beginning. Since clinical examination alone cannot reliably differentiate advanced in situ AKs from early invasive SCC, this may lead to a misclassification bias and overestimation of the prognostic value of clinical grading. The observational findings of our studies do not support the link between high Olsen grade with parameters of invasive risk. Instead of the Olsen grade, pain upon palpation was the most predictive non-interventional clinical parameter of histological risk factors for invasion. However, the biggest limitation of non-longitudinal observation in biopsy or excision of AK cannot be overcome with conventional histopathology. To bridge the gap between clinical assessment and histological risk factors, advancements in non-invasive imaging technologies offer potential avenues. Among imaging methods are confocal laser scanning microscopy (CLSM), optical coherence tomography (OCT), and line-field confocal optical coherence tomography (LC-OCT) [22]. The disadvantage of CLSM is usually the insufficient penetration depth to evaluate the dermoepithelial junction, especially in the case of basal proliferating AK. Traditional OCT had higher penetration depth than CLSM but lacked image quality to effectively evaluate cell borders. With LC-OCT, sufficient penetration depth and image quality were introduced, benefiting the clinical evaluation of AK with non-invasive imaging techniques. Recent studies have demonstrated that LC-OCT can effectively assess the PRO score of AKs in vivo, providing real-time non-invasive insights into the basal proliferation patterns [30]. Ruini et al. showed that LC-OCT imaging correlates well with histological findings, enabling non-invasive evaluation of downward proliferating atypical keratinocytes in AKs [30]. Furthermore, the integration of artificial intelligence with LC-OCT has enhanced the accuracy and efficiency of PRO score assessments. Recent studies have demonstrated that convolutional neural networks can automatically quantify PRO scores from LC-OCT images with high concordance to expert evaluations [31,32]. This AI-based approach not only streamlines the assessment process but also reduces interobserver variability, offering a standardized method for evaluating AKs. The application of such technologies in clinical practice could facilitate more precise monitoring of lesion progression and treatment response.

The findings of our retrospective evaluation suggest that the commonly used Olsen classification may not reliably reflect biologically aggressive features of AKs and should therefore not be solely relied upon for risk stratification in clinical decision-making. However, limitations must be considered when interpreting these findings. One inherent limitation of this and similar studies is that once a lesion is excised for histopathological analysis, it cannot be followed longitudinally. This limits the ability to directly associate morphological features with conventional histopathologic diagnosis with actual clinical progression [8]. This issue also applies to previous studies reporting increased progression risk in Olsen grade III lesions, where it often remains unclear whether the lesions were already invasive at the time of excision, potentially leading to misclassification [29].

## 5. Conclusions

In conclusion, our findings demonstrate that clinical hyperkeratosis grading using the Olsen system does not reliably predict histological basal proliferation patterns in AKs. In contrast, the presence of pain may serve as a useful clinical marker to identify biologically active lesions. Emerging technologies such as LC-OCT, particularly in combination with AI-based image analysis, offer promising tools for non-invasive PRO score assessment for routine clinical assessment of histologic risk factors and longitudinal individual lesion observation. Together, these insights support a more integrated approach to risk stratification, combining clinical observation, patient-reported symptoms, and the outlook of using advanced imaging to guide the management of actinic keratoses.

## Figures and Tables

**Table 1:** Demographic, clinical, and histologic characteristics (*n* = 380).

Characteristic	Category	*n* (%)
Sex		
	Male	297 (78.2)
	Female	83 (21.8)
Age		
	Years	74.1 (9.5) *
Location of lesion		
	Face and forehead	219 (57.6)
	Balding scalp and neck	128 (33.7)
	Other	33 (8.7)
Olsen grade		
	1	114 (30.0)
	2	194 (51.1)
	3	72 (18.9)
PRO stage		
	I	131 (34.5)
	II	103 (27.1)
	III	146 (38.4)
AK stage		
	I	84 (22.1)
	II	167 (43.9)
	III	129 (33.9)
Painful upon palpation		
	No	259 (68.2)
	Yes	121 (31.8)

* Data are mean (standard deviation).

**Table 2:** Presence of histomorphological variants across Olsen grades.

Variant	Present	Olsen 1 (*n* = 86)	Olsen 2 (*n* = 141)	Olsen 3 (*n* = 47)	*p*-Value *	Pairwise Comparisons **
Acantholytic						
	No	71 (82.6%)	126 (89.4%)	37 (78.7%)		
	Yes	15 (17.4%)	15 (10.6%)	10 (21.3%)	0.121	
Atrophic						
	No	84 (97.7%)	139 (98.6%)	46 (97.9%)		
	Yes	2 (2.3%)	2 (1.4%)	1 (2.1%)	0.851	
Bowenoid						
	No	84 (97.7%)	140 (99.3%)	46 (97.9%)		
	Yes	2 (2.3%)	1 (0.7%)	1 (2.1%)	0.498	
Hyperkeratotic						1 vs. 2: 0.264
	No	78 (90.7%)	114 (80.9%)	24 (51.1%)		1 vs. 3: *p* < 0.001
	Yes	8 (9.3%)	27 (19.1%)	23 (48.9%)	0.001	2 vs. 3: *p* < 0.001
Lichenoid						
	No	85 (98.8%)	134 (95.0%)	45 (95.7%)		
	Yes	1 (1.2%)	7 (5.0%)	2 (4.3%)	0.347	
Pigmented						
	No	85 (98.8%)	138 (97.9%)	47 (100%)		
	Yes	1 (1.2%)	3 (2.1%)	0 (0%)	0.826	

* Fisher’s exact test. ** Pairwise comparison of Olsen groups (Fisher’s exact test).

**Table 3:** Frequency of Pain in Relation to Clinical Grading and Histopathological Features of AKs.

		Painful Lesions				
Classification	Grade/Feature	*n*	No	Yes	*p*-Value *	Pairwise Comparisons **
Olsen						
	1	114	79 (69.3%)	35 (30.7%)		
	2	194	134 (69.1%)	60 (30.9%)		
	3	72	46 (63.9%)	26 (36.1%)	0.690	
PRO						
	I	131	101 (77.1%)	30 (22.9%)		1 vs. 2: 0.232
	II	103	72 (69.9%)	31 (30.1%)		2 vs. 3: 0.084
	III	146	86 (58.9%)	60 (41.1%)	0.005	1 vs. 3: 0.001
AK						
	I	84	65 (77.4%)	19 (22.6%)		
	II	167	110 (65.9%)	57 (34.1%)		
	III	129	84 (65.1%)	45 (34.9%)	0.115	
Histopathological variants						
Acantholytic						
	No	234	187 (79.9%)	47 (20.1%)		
	Yes	40	25 (62.5%)	15 (37.5%)	0.023	
Atrophic						
	No	269	209 (77.7%)	60 (22.3%)		
	Yes	5	3 (60.0%)	2 (40.0%)	0.317	
Bowenoid						
	No	270	210 (77.8%)	60 (22.2%)		
	Yes	4	2 (50.0%)	2 (50.0%)	0.221	
Hyperkeratotic						
	No	216	169 (78.2%)	47 (21.8%)		
	Yes	58	43 (74.1%)	15 (25.9%)	0.486	
Lichenoid						
	No	264	204 (77.3%)	60 (22.7%)		
	Yes	10	8 (80.0%)	2 (20.0%)	1.000	

* Fisher’s exact test. ** Pairwise comparisons of groups (Fisher’s exact test).

## Data Availability

Study data can be accessed upon request to the corresponding author.

## Abbreviations

The following abbreviations are used in this manuscript:

AK	Multidisciplinary Digital Publishing Institute
AK I–III	Histological grading of upward proliferation according to Röwert-Huber et al. (AKI–III)
PRO I–III	Histological grading of basal proliferation according to Schmitz et al. (PRO I–III)
AKASI	Actinic Keratosis Area and Severity Index
κ	Cohen’s kappa coefficient
ρ	Spearman’s rank correlation coefficient
cSCC	Cutaneous squamous cell carcinoma
H&E	Hematoxylin and eosin
UV	Ultraviolet
CLSM	Confocal laser scanning microscopy
OCT	Optical coherence tomography
LC-OCT	Line-field confocal optical coherence tomography
